# The missing link in FAIR data policy: biodata resources in life sciences

**DOI:** 10.1038/s41597-026-06690-w

**Published:** 2026-02-06

**Authors:** Lucy Poveda, Gavin Farrell, Silvio C. E. Tosatto, Monique Zahn-Zabal, Patrick Ruch, Julien Gobeill, Robert M. Waterhouse, Christophe Dessimoz

**Affiliations:** 1 SIB Swiss Institute of Bioinformatics, Lausanne, Switzerland; 2 Department of Biomedical Sciences, University of Padova, Padova, Italy; 3 Institute of Biomembranes, Bioenergetics and Molecular Biotechnologies, National Research Council (CNR-IBIOM), 70126 Bari, Italy; 4 Information Sciences, HES-SO/HEG Geneva, 1227 Carouge, Switzerland; 5 University of Lausanne, Lausanne, Switzerland

**Keywords:** Databases, Funding, Research management, Data integration

## Abstract

In the life sciences, FAIR principles have reshaped research policy, but their implementation still relies largely on individual researchers – many of whom lack the expertise or support needed to make data truly reusable. Realising FAIR’s promise requires sustained investment in the infrastructures that organise, standardise, and curate data: deposition databases and knowledgebases. These biodata resources are especially critical for AI, which depends on large, high-quality, and consistent data. Landmark advances like AlphaFold and the COVID-19 response illustrate how sustained curation and standardisation in expert resources such as UniProt and the Protein Data Bank have enabled rapid innovation. Yet biodata resources remain precariously funded, jeopardising long-term sustainability and the expert workforce they require. To support ambitious, data-driven science, funders must align policy and budgets by establishing dedicated mechanisms that allocate a small (e.g., 1%), but strategic and stable share, of research funding to core data infrastructures. This would maximise the value of public investment, strengthen open science and international collaboration, and unlock the full potential of FAIR.

## Introduction

Over the past decade, the FAIR principles^1^ which provide guidance in making data Findable, Accessible, Interoperable, and Reusable have transformed the way research is funded and evaluated. Funders now routinely require data management plans, researcher training, and data deposition. Less attention, however, has been given to the infrastructure that enables FAIR in practice: the biodata resources – deposition databases and knowledgebases – that turn scattered data sets into readily-available coherent knowledge.

Without infrastructure, FAIR data policy risks becoming a compliance exercise where data are shared but remain fragmented, inconsistently annotated, or practically inaccessible. Achieving FAIR and reaping its benefits for discovery, artificial intelligence (AI), and innovation depends on infrastructure designed to capture, curate, and connect research data systematically.

This article argues that investing in biodata resources is one of the most effective and cost-efficient ways of achieving the FAIR principles. It calls on funders to provide stable, competitive support for these vital resources on the order of ~1% of research budgets^2,3^ and maximise the impact of public investment in science^4^.

## FAIR principles in the era of AI

The FAIR principles ensure that data can be found, accessed, and understood by both humans and machines^1^. In the era of big data and AI, FAIR is more essential than ever because AI performance scales with data quality and consistency.

AI-driven science needs broad availability of structured knowledge, not just data^5^. For example, AlphaFold’s revolutionary, AI-based solution to the protein folding problem^6^ and recognised by the 2024 Nobel Prize in Chemistry was possible due to decades of work to represent protein structure models in consistent, machine-readable formats. This AI model did not trawl through disparate lab reports, papers, or general purpose data repositories; it learned from the Protein Data Bank (PDB), which contains consistent 3D protein models and rich metadata^7,8^, and UniProt, an expertly-curated knowledgebase of protein sequences, functional annotations, and cross-references to other biomolecular databases^9^.

These resources provide stable identifiers, semantic standards, and relationships between sequences and structures – all essential for robust AI generalisation.

## COVID-19 showed how biodata resources turn data into impact

The COVID-19 pandemic offers another striking case study of the role and impact of biodata resources. The rapid publication of the first SARS-CoV-2 genome sequence in January 2020^10^ is rightly celebrated as a triumph of data sharing. But what happened next was critically dependent on long-standing open biodata resources.

Researchers interpreted the viral sequence rapidly because UniProt and ViralZone^11^ provided curated coronavirus knowledge. Comparison with related sequences in the European Nucleotide Archive (ENA)^12^. The structure of the spike protein, critical for vaccine development, was modeled within days using SWISS-MODEL^13^, drawing on public structural data and annotation frameworks. Nextstrain^14^, integrating new genome sequences with global surveillance data.

Despite this impact, the foundational curation and infrastructure largely went unnoticed and remain precariously funded.

## FAIR, not bare: the role of deposition databases and knowledgebases

Storing data securely and ensuring its long-term availability for future use is an essential aspect of the research data life cycle. Deposition databases, such as GenBank^15^ or ENA for nucleotide sequences and the Electron Microscopy Public Image Archive^16^ for raw electron microscopy images, preserve primary datasets with basic metadata, persistent unique identifiers, and search interfaces. Generalist repositories such as Zenodo^17^ address broader needs.

Knowledgebases such as PomBase^18^ Rhea^19^ and Bgee^20^, all build on these primary data to produce curated, connected information. According to ELIXIR’s definitions, archival/deposition repositories take in *de novo* data from scientists, whereas knowledgebases “add substantial value through expert curation, annotation of metadata, sophisticated data processing and data integration”^21^. Knowledgebases build on deposition databases but go significantly further by adding layers of expert curation, semantic integration, and biological interpretation. Their core functions focus on transforming dispersed experimental data and scientific papers into coherent, machine- and human-readable knowledge. In practice, many leading biodata resources even blur the line and do both archive and knowledge building as BioStudies^22^.

While both deposition databases and knowledgebases contribute to the FAIRification of life science data, they play distinct and complementary roles within the data ecosystem (Table 1).

**Table 1:** Contributions to data FAIRification by the two kinds of biodata resource infrastructure.

Deposition databases	Knowledgebases
• Persistent identifiers: Assigning stable, unique accession numbers (e.g., GenBank/ENA accession numbers for sequences, PDB identifiers for experimental protein structures) or in few cases DOIs as in ModelArchive^41^, making data reliably findable and citable. • Basic quality control: Enforcing minimum metadata requirements and standard file formats at the point of submission, ensuring deposited data meet community-agreed standards of completeness and structure. • Standardisation of formats: Mandating common syntactic and semantic standards (e.g., FASTA, VCF, mmCIF, JATS), thus facilitating data parsing and exchange. • Long-term preservation: Providing technical infrastructure, ensuring the accessibility of data over time. • Discoverability tools: Providing search interfaces and APIs, allowing users/machines to locate and retrieve relevant datasets.	• Expert curation & quality control: Evaluating, annotating, and integrating evidence from multiple sources (often including literature), ensuring accuracy, consistency, and biological relevance far beyond minimal deposition standards. • Semantic standardisation & ontologies: Co-developing and applying controlled vocabularies and community ontologies (e.g., Gene Ontology, Disease Ontology) to describe biological concepts, enabling interoperability not just at the data format level, but at the semantic level. • Cross-referencing & integration: Linking diverse data types (genes, proteins, pathways, phenotypes, literature) and identifiers across databases, creating rich knowledge graphs that reflect biological relationships. • Aggregation & synthesis: Combining evidence across species, experiments, and data types, providing consensus views or generalised knowledge (e.g., GO annotations summarising gene functions across organisms). • Search and analysis tools: Providing user-friendly interfaces, programmatic access, and visualisations, facilitating discovery and reuse of curated knowledge. • Community training & support: Offering detailed documentation, tutorials, and help desks guiding users in data deposition, access, and reuse, thereby fostering best practices across the research community.

## Curation and curation-support infrastructure

Curation is an expert driven task requiring specialised personnel and a supporting ecosystem of IT and semantic tools.Further, curation is directly dependent on the availability of publications, whose access has been greatly enhanced thanks to Open Access libraries such as EuropePMC^23^ or SIBiLS/BiodiversityPMC^24^. Curated databases were also pioneers in exploring how AI-driven search engines – today’s Retrieval Augmented Generation can support their curation workflows^25^. Today, these databases are evolving towards delivering accurate data description workflows^26^ and schemas, supported with semantically unambiguous descriptors (e.g., accession numbers, ontological concepts^27^, to deliver a high-density data net tailored to directly drive AI developments.

## Knowledgebases as critical enablers of AI breakthroughs

Life science knowledgebases deserve, indeed, special attention in the context of AI developments. By transcending individual experiments, they create a consensus view of biology and make it available through machine-readable formats and interfaces. This integrated, consistent, and quality-controlled layer of distilled knowledge is a layer above the raw data and exactly what AI algorithms need for effective training and testing. An AI trying to reason about gene function or disease mechanisms benefits enormously from the existence of a curated knowledge graph, rather than having to infer connections from scattered experimental datasets. Building such knowledge representations through synthesis and consensus has become an increasingly important role in science, filled by expert biocurators in projects like Gene Ontology and UniProt. The resulting knowledgebases make the latest knowledge broadly usable beyond the original subfield and available for computational exploitation^5^. In essence, knowledgebases convert the deluge of specialised data into an organised, interoperable knowledge network, paving the road for AI-driven discoveries.

AI and knowlegbases are being used in a range of applications. PomBase^18^ integrates information about Schizosaccharomyces pombe (fission yeast) through expert literature curation such as genome sequences, gene functions and mutant phenotypes, thus serving as a reference for a study applying phenomics and machine-learning approaches^28^. The team providing Rhea^19^, an expert-curated knowledgebase of chemical and transport reactions of biological interest, also generated EnzChemRED (Enzyme Chemistry Relation Extraction Dataset), a new training and benchmarking dataset to support the development of Natural Language Processing (NLP) methods^29,30^. Bgee takes gene expression data (from RNA-seq, *in situ* hybridisation, etc.) from many different species and conditions, and curates them into a consistent baseline of where genes are expressed, using anatomy ontologies and rigorous quality checks. They are currently developing a pilot implementation of annotation of single-cell RNA-Seq data guided by AI.

## The complementary roles of researchers and biodata resources

Current efforts to achieve FAIR principles heavily emphasise the role of individual researchers. But expecting every lab to become expert in metadata standards, ontologies, and long-term data curation is unrealistic and inefficient. Researchers are trained to innovate at the frontier of science; asking them to also master data archiving and interoperability standards for each dataset diverts their time and efforts, as well as often resulting in suboptimal outcomes. For instance, inconsistent metadata or under-annotated datasets, while technically “available,” are hard to reuse.

Centralised resources can achieve these aspects of FAIRification more efficiently. Biodata resource staff specialise in data curation and interoperability; they can perform these tasks at scale, with higher expertise and automation level, while still maintaining quality requirements. When deposition databases enforce standard formats and metadata upon submission, they automatically improve consistency and findability. Knowledgebase curators, in turn, devote focused effort to refining and linking data, a job that would be prohibitively laborious if every lab attempted to do so in isolation. Even labour-intensive expert curation is a small fraction of the cost of generating the data in the first place and increases data value many-fold^5^.

## Funding challenges and the case for sustained investment

Even as biodata resources become central to science and innovation, their funding remains precarious. Millions of life scientists rely on bioinformatics databases, especially high-value curated knowledgebases, yet most of these resources lack secure, long-term support and depend on short-term grants misaligned with their mission^31^. Treated as conventional research projects rather than critical infrastructure, they suffer chronic underfunding and the loss of highly specialised personnel. Unlike individual research projects, which can sometimes be paused with limited consequences, biodata resources underpin entire networks of dependent users, services, and tools. Interruptions in their operation reverberate widely, making their stability essential.

The Global Biodata Coalition’s list of Global Core Biodata Resources (GCBRs)^32^ responds to these concerns by identifying databases “of fundamental importance to the wider life-science community… and the long-term preservation of biological data,” whose loss would have “a highly detrimental impact on the global research endeavour”. Yet this recognition has not translated into new funding instruments or increased budgets. Despite rising usage and widespread endorsement of FAIR by funders, overall funding for biodata resources has stagnated or declined in real terms. The Wellcome Trust has discontinued competitive biodata resource funding, and funding through national instruments (notably NIH, BBSRC or Swiss Secretariat for Education, Research, and Innovation) has not kept up with inflation or demand, leading several Global Core Biodata Resources to lose major funding sources.

This is paradoxical, because sustaining the most impactful biodata resources is comparatively affordable. Gabella *et al*.^2^ proposed an “Infrastructure Model” in which agencies reserve a fixed share of grant budgets to support core data resources in a stable, competitive way. Their estimates suggest that less than 1% of life science research funding would suffice to cover the worldwide costs of core data resources for knowledgebases and deposition archives. This is a remarkably small fraction to ensure that the most important data and knowledge produced can be properly preserved and made FAIR. Given their outsized impact – both in safeguarding past research investments and accelerating new discoveries – supporting these resources is arguably among the highest-return uses of research funding.

## Aligning responsibilities, recognition, and incentives

Achieving and sustaining FAIR data is a shared responsibility. Researchers who generate data have a responsibility to organise and deposit their data in appropriate repositories with the appropriate metadata. Data resource teams (curators, software engineers, data scientists, data stewards) take on the responsibility of maintaining and enhancing these data for the community. Funders and research institutions, in turn, carry the responsibility of enabling this ecosystem through competitive funding and monitoring, policy, and recognition.

Such division of labour requires policy changes and cultural shifts in how we evaluate and reward contributions in science. Maintaining a widely-used database or curating an ontology can be just as vital to scientific progress as authoring a high-profile paper, yet traditional academic reward systems often undervalue such contributions. Here, the emerging principles of initiatives like CoARA (Coalition for Advancing Research Assessment)^33^ are highly pertinent. CoARA calls for a broad recognition of the diversity of contributions to, and careers in, research and explicitly seeks to integrate the recognition of contributions to open science – including data sharing and infrastructure – in research assessment.

Funders must recognise biodata resources not as a “burden”^34^ or an expenditure “to the detriment of new research”^35^, but as core scholarly activities integral to fulfilling their mission. In Europe, initiatives like EOSC have recognised the critical importance of issues such as defining trustworthy repositories, ensuring long-term data preservation, establishing a transdisciplinary expert curation network, and improving data discoverability. Currently, these topics are addressed within short-term projects, typically lasting only three to four years, such as FIDELIS, EOSC EDEN^36^, and EOSC Data Commons^37^. However, these projects do not fund the maintenance of the underlying deposition databases and knowledgebases they depend on; rather, they build upon existing resources.

To ensure continuity and impact, the world needs dedicated, long-term funding instruments for biodata resources guided by the same principles of excellence, impact, CARE principles (Collective Benefit, Authority to Control, Responsibility, Ethics) and competitiveness that apply to research grants, but tailored to the specific role of resources e.g. Ref. ^38^ would provide both the clear signalling and sustained investment needed to make FAIR a reality in practice. Importantly, prioritisation should not reinforce incumbency: assessments must consider the scientific importance of the data, the degree of community dependence, and the cost–benefit profile of each resource. Evaluating both large, widely used infrastructures and specialised niche resources on their actual value to the research ecosystem – rather than on scale alone – would support a balanced, high-performing landscape. This would strengthen collaboration between researchers and data curators and ensure that knowledgebases keep pace with scientific demand.

## Securing FAIR’s missing link and science’s return on investment

In summary, FAIR data is the fuel driving modern life science research and innovation^39^, and its importance is only amplified by the rise of AI and data-driven discovery. While individual researchers are encouraged to manage and share their data, we must find a way to support the critical role of data resources, the deposition databases and knowledgebases that actually make data findable, accessible, interoperable, and reusable in practice. These resources perform the heavy lifting of data standardisation, integration, and preservation and turn isolated experimental results into collective scientific knowledge. They exemplify how democratising data increases its value: once data is integrated into a curated knowledgebase, they can serve the entire community and spark new discoveries well beyond the original lab’s research intentions and individual subfields. They make it possible to turn a newly sequenced virus genome into highly accurate PCR tests within days, vaccines within months, and a flood of fragmented sequences into real-time global surveillance to address global pandemics and enable science-based policies.

The return on investment in such resources is extraordinarily high^40^, yet their funding and recognition has lagged behind their impact. It is time for funders and institutions to treat biodata resources as essential infrastructure, despite their digital nature, as they are equally if not more important than physical laboratories and instrumentation. We strongly encourage them to be recognised, supported and financed accordingly. Dedicating in the order of 1% of research funding to core data resources would ensure that the outputs of the other 99% are properly conserved and made maximally useful. Moreover, embracing a holistic view of scientific contribution in line with CoARA will foster an environment where data sharing and curation are rewarded, not neglected.

In the quest for effective cures, sustainable agriculture, environmental solutions, and fundamental insights into life, data resources are our knowledge vaults and launchpads. Strengthening these resources is a direct investment in the future of FAIR, open, and AI-enabled science – an investment our research ecosystem cannot afford to shortchange.
